# Ethnic/geographic analysis Of Human T-Lymphotropic Virus type 1 (HTLV-1) infection among Buenos Aires residents in Argentina

**DOI:** 10.1186/1742-4690-8-S1-A88

**Published:** 2011-06-06

**Authors:** María Eirin, Claudio Bravi, Leandro Jones, Carolina Berini, Cecilia Delfino, Mirna Biglione

**Affiliations:** 1 Centro Nacional de Referencia para el SIDA, Facultad de Medicina, UBA, Buenos Aires, Argentina; 2 Instituto Multidisciplinario de Biología Celular, La Plata, Buenos Aires, Argentina; 3 División de Biología Molecular, Estación de Fotobiología Playa Unión, Playa Unión, Chubut, Argentina

## Introduction

HTLV-1 is endemic in the Northwest among Kolla natives while Buenos Aires city (BA) is considered as non-endemic. In the last decades, migrations to BA have changed from Europeans to Latin-Americans from countries with endemic focus for HTLV-1 infection (Bolivia, Paraguay, Peru, Brazil, and Chile) and also from different African countries.

## Objective

To determine the phylogeographic origin of HTLV-1 strains and the ethnic background of HTLV-1 positive residents of Buenos Aires.

## Materials and methods

63 HTLV-1 positive BA residents were retrospectively studied. Phylogeny of LTR region was performed by PAUP 4.0 and tree topology was visualized by TreeView. The ethnic origin of individuals was inferred by the study of mitochondrial DNA haplogroups (complete control region).

## Results

All LTR sequences were classified as Cosmopolitan subtype/Transcontinental subgroup. Twenty-five of them, belonging to individuals of Amerindian and 3 of African ancestry, were included in the Big Latin American cluster with HTLV-1 references from Amerindians. On the other hand, 9 LTR sequences belonging to individuals of Amerindian ancestry, grouped with references from people of black ethnic component of Africa, French Guiana and Suriname (Small Latin American and South African clusters).

## Conclusions

This study confirms the presence HTLV-1 cosmopolitan subtype/ Transcontinental subgroup in BA residents and shows HTLV-1 strains from individuals of Amerindian ancestry closely related to references from Africa and Latin-American countries with an important afro-amerindian ethnic component. Finally, these results highlight the importance of ethnic origin of HTLV-1infected people and the retroviral phylogeny to understand the influence of migration waves in the dynamics of the infection in a specific geographic area.

